# Cardiac substructure dosimetry in postoperative breast-conserving radiotherapy: a novel 8-field IMRT approach for internal mammary node irradiation using MONACO

**DOI:** 10.3389/fonc.2025.1681699

**Published:** 2026-01-20

**Authors:** Ziyi Xie, Shuai Hao, Xiao Wu, Yinliang Liu, Chaoen Bao, Ruhan Zhao, Ming Liu, Xiaohui Cao

**Affiliations:** 1 Department of Oncology, Hebei Medical University Third Hospital, Shijiazhuang, China; 2 Center of Oncology, Aerospace Center Hospital, Beijing, China

**Keywords:** breast cancer, cardiac substructures, dosimetry, intensity-modulated radiotherapy, internal mammary lymph nodes

## Abstract

**Objective:**

To evaluate the dosimetric impact of internal mammary lymph node (IMN) inclusion versus exclusion (non-IMN) on cardiac substructures in postoperative breast-conserving radiotherapy, providing evidence-based insights for clinical decision-making.

**Methods:**

This study included 20 breast cancer patients (10 on the left and 10 on the right) who had previously received radiotherapy in our hospital after breast conserving surgery. The clinical target volume (CTV) encompassed the ipsilateral breast, supraclavicular lymph nodes, and internal mammary lymph nodes (IMNs). Organs at risk (OARs) comprised the heart and its substructures—including the left ventricle (LV), left atrium (LA), right ventricle (RV), right atrium (RA), anterior myocardial territory (AMT), left anterior descending artery (LAD), left circumflex artery, and right coronary artery—as well as bilateral lungs, ipsilateral/contralateral lungs, contralateral breast, thyroid, and spinal cord. For both target delineation strategies, treatment planning utilized 8-field fixed-beam intensity-modulated radiation therapy (IMRT) with 6 MV X-rays, delivering 50 Gy in 25 fractions over 5 weeks to 95% of the planning target volume (PTV). Continuous variables were reported as mean ± standard deviation (SD). Normality was assessed using Shapiro-Wilk tests, with paired t-tests applied for normally distributed data and Wilcoxon signed-rank tests for non-parametric comparisons. Statistical significance was defined as P<0.05 (two-tailed).

**Results:**

Comparative dosimetric analysis revealed significantly improved planning target volume homogeneity index (HI) and conformity index (CI) in the non-IMN treatment irradiation cohort compared to the IMN group for both left- and right-sided breast cancers (P< 0.05). Subgroup analysis: Left-sided breast cancer analysis: The IMN-irradiated cohort demonstrated significantly elevated cardiac dose parameters, with increased Dmax (P<0.05) and Dmean (P<0.05) for the whole heart compared to non-IMN treatment. Paradoxically, the left ventricle exhibited reduced mean dose (707.61 ± 141.28 cGy vs. 825.94 ± 141.46 cGy, P<0.05) in the IMN group. Significant dose escalation was observed in the right cardiac structures, including right ventricle Dmax/Dmean, right atrium Dmean, anterior myocardial territory Dmax, and right coronary artery Dmax/Dmean (P<0.05). However, no statistically significant differences were detected in heart volumetric parameters (V5, V10, V40), left ventricle Dmax, left atrial doses (Dmax/Dmean), right atrium Dmax, anterior myocardial territory Dmean, or coronary artery doses (LAD and left circumflex Dmax/Dmean). For right-sided breast cancer cases, comparative dosimetric analysis revealed distinct patterns in cardiac substructure exposure: Cardiac dose parameters: No statistically significant differences were observed in mean heart dose (Dmean) or low-dose exposure (V5) between the treatment groups. Atrial exposure: The IMN-irradiated cohort demonstrated significantly elevated maximum dose (Dmax) to the right atrium (P< 0.05). Myocardial territory: The IMN group showed increased maximum dose (P< 0.05) and significantly higher V10 (P<0.05) to the anterior myocardial territory. Coronary arteries: A paradoxical reduction in left circumflex artery Dmax was observed in the IMN-treated patients (P<0.05). Non-significant parameters: Anterior myocardial territory Dmean, left circumflex artery Dmean, LAD (both Dmax/Dmean), and right coronary artery (both Dmax/Dmean) showed comparable dose distributions between groups.

**Conclusion:**

1. The dosimetric analysis reveals that 8-field intensity-modulated radiation therapy (IMRT) with internal mammary node (IMN) irradiation significantly increases mean heart dose and critical cardiac substructure exposure in left-sided breast cancer (P<0.05), potentially elevating long-term cardiovascular toxicity risks. However, right-sided treatments demonstrated no significant differences in mean heart dose (P>0.05) or left anterior descending artery (LAD) dose exposure. These findings suggest that clinical decision-making should carefully weigh the competing risks of locoregional recurrence against potential cardiotoxicity, with consideration given to advanced radiotherapy techniques for cardiac dose optimization when treating left-sided malignancies. 2. Current evaluation metrics for breast cancer radiotherapy planning, predominantly focused on mean heart dose and low-dose volume parameters, may not sufficiently capture the risk of radiation-induced cardiac toxicity. Comprehensive dosimetric assessment requires delineation of cardiac substructures (e.g., ventricles, coronary arteries) as discrete organs-at-risk (OARs), with their dose constraints systematically integrated into plan optimization and quality assurance protocols. This paradigm shift toward substructure-sparing approaches could enhance the therapeutic ratio by minimizing late cardiovascular complications while maintaining target coverage. 3. Postoperative breast-conserving radiotherapy should follow guideline recommendations regarding IMN irradiation strictly.

## Introduction

According to the latest global cancer statistics (1), breast cancer represents the most prevalent malignancy worldwide, with approximately 2.3 million new cases diagnosed in 2022 - surpassing lung cancer (2.2 million cases) as the most commonly occurring cancer. Notably, breast cancer ranks as the fourth leading cause of cancer-related mortality globally.

Postoperative radiotherapy following breast-conserving surgery has emerged as standard-of-care treatment, demonstrating dual oncologic benefits: significant reduction in both local recurrence risk and breast cancer-specific mortality for early-stage disease (2). Furthermore, regional nodal irradiation provides survival advantages for patients with high-risk features (3). Paradoxically, contemporary evidence reveals a 1.7-fold elevated risk of cardiac mortality among radiotherapy recipients compared to surgery-only counterparts, underscoring the critical need for cardiac-sparing techniques in modern radiation oncology practice (4–6). A linear dose-response relationship exists between cardiac radiation exposure and coronary risk, with each 1 Gy increase in mean heart dose elevating the probability of major coronary events by 7.4% (4). The heterogeneous dose distribution characteristic of breast radiotherapy frequently results in focal dose maxima within the anterior cardiac compartment, particularly affecting the left anterior descending coronary artery (LAD). Current radiobiological evidence confirms exceptional radiation sensitivity of coronary vasculature, with particular clinical significance for the LAD as both the primary myocardial perfusion source and the most common anatomical site of radiation-associated ischemic heart disease (7). The Early Breast Cancer Trialists’ Collaborative Group (EBCTCG) meta-analysis of multiple prospective studies demonstrated that while radiotherapy reduced breast cancer recurrence and mortality, it concurrently increased non-cancer mortality, resulting in limited overall survival benefit (8). These findings reflect treatment outcomes from the 1970s-1980s, when radiotherapy was predominantly delivered using two-dimensional techniques prior to the widespread adoption of precision radiotherapy. During this era, the medical community’s understanding of radiation-induced cardiotoxicity in breast cancer treatment remained incomplete.

As radiotherapy techniques continue to advance and cancer patient survival progressively extends, the emerging field of cardio-oncology has gained prominence, with cardiac substructure research now at the forefront. This study aims to evaluate the dosimetric impact of two target delineation approaches—Internal Mammary Lymph Node (IMN) inclusion versus exclusion (non-IMN)—on cardiac substructures during post-breast-conserving surgery radiotherapy, thereby providing critical evidence to inform clinical decision-making.

## Materials and methods

### Inclusion and exclusion criteria

This study included 20 patients with breast cancer after breast-conserving surgery who had received radiotherapy in our hospital (10 cases on the left and right sides). All patients completed the whole cycle of adjuvant radiotherapy according to the doctor‘s advice.

Inclusion criteria:

- Age ≥ 18 years old.
- Preoperative bilateral breast MRI, B-ultrasound or mammography confirmed that the primary lesion was unilateral.
- Unilateral breast conserving surgery was performed, and postoperative pathology confirmed breast cancer: (including invasive breast cancer and breast ductal carcinoma *in situ*, etc.).
- no history of malignant tumor.
- Patients after breast-conserving surgery have postoperative radiotherapy indications.
- Exclusion criteria:
- pregnant and lactating women.
- patients with other tumor history or recurrence after breast conserving therapy.
- received breast reconstruction.
- incomplete medical records.

### Methods

#### Patient positioning and immobilization protocol for breast radiotherapy

All patients were positioned supine with arms crossed above the head and elbows supported, with lead wires marking the chest wall incision and drainage port for reference during simulation using a Siemens 128-slice spiral CT scanner, acquiring 5-mm thick slices from 2 cm above the cricothyroid membrane to the inferior thoracic margin. The clinical target volumes (CTVs) were contoured according to American College of Radiation Oncology (ACRO) guidelines as follows: (1) The chest wall CTV encompassed the region from the inferior border of the clavicular head superiorly to the level of breast tissue loss inferiorly on CT imaging, bounded anteriorly by the skin surface (with surgical scars marked by wire), posteriorly by the costal pleura, laterally by the midaxillary line (excluding the latissimus dorsi muscle), and medially by the ipsilateral parasternal line; (2) The supraclavicular nodal CTV extended from the inferior border of the cricoid cartilage superiorly to the caudal edge of the clavicular head inferiorly, delineated anteriorly by the anterior margin of the scalene muscles, posteriorly excluding the thyroid gland and trachea, laterally by the lateral border of the sternocleidomastoid muscle (incorporating the first rib-clavicular junction), and medially by the medial border of the sternocleidomastoid muscle. All contours were reviewed and approved by a radiation oncologist with breast subspecialty expertise. (3) The internal mammary lymph node CTV (CTV-IMN) was defined with the superior border at the first rib’s upper edge, inferior border at the fourth rib’s upper edge, lateral margin extending 4 mm beyond the internal mammary vessels, and medial margin 4 mm medial to the vessels. The internal mammary planning target volume (PTV-IMN) included a 5 mm isotropic expansion of CTV-IMN (excluding lung tissue), while the non-internal mammary PTV (PTV-nonIMN) excluded this region. All PTVs received a 5 mm 3D expansion, with skin-facing surfaces retracted 5 mm subdermally. Organs at risk comprised the heart and its substructures (left/right ventricles, left/right atria, precordium, left anterior descending artery, left circumflex artery, right coronary artery), bilateral lungs, contralateral breast, thyroid, and spinal cord. A single radiation oncologist performed all delineations, verified by two senior radiation oncologists to ensure protocol compliance.

#### Prescription dose and plan design

This study used a patient-controlled design. We divided patients with right breast cancer and left breast cancer into independent analysis subgroups. For each subgroup, based on the same image data set, we calculated and compared the dosimetric parameters of the two radiotherapy plans with and without the internal mammary lymphatic drainage area.The two target areas were treated with 8-field IMRT 6MV-X radiotherapy technology, and the prescription dose of PTV was 50Gy/25f/5W. The normalization method was that at least 95% of PTV met the prescribed dose, and the normal tissue dose limits included: V5< 50%, V20< 30%, V30< 20%, D_mean_< 12 Gy in the affected lung, D_max_< 10 Gy in the healthy breast; left breast cancer: heart D_max_< 50 Gy, D_mean_< 7.5 Gy.

Right breast cancer: heart D_max_< 50 Gy, D_mean_< 4.0 Gy, other tissues such as spinal cord, trachea, ipsilateral humeral head should be avoided as much as possible when setting the field, to avoid direct exposure to the heart.

#### Evaluating indicator

Evaluation parameters of PTV: PTV evaluation parameters: maximum dose (D_max_), mean dose (D_mean_), V95%, V107%, D2%, D90%, D95%, conformity index (CI), homogeneity index (HI), monitor units (MU). CI was the ratio of prescribed isodose volume (PIV) to PTV. Homogeneity index HI formula: HI = (D2% -D98%)/(D50%) (D2% represents the dose received by 2% of the target volume on the dose volume histogram, D98% represents the dose received by 98% of the target volume on the histogram, D50% represents the dose received by 50% of the target volume on the dose volume histogram).

Note: The closer the HI value is to 0, the better the uniformity of the plan is.

The formula of conformal index is: $\text{CI}=\frac{V_{Rx}}{{\text{V}}_{\text{T}}}\times \frac{V_{Rx}}{V_{RI}}$. V_Rx_ represents the volume of the target area irradiated by the prescription dose, V_T_ represents the volume of the entire target area, and V_RI_ represents all the volumes irradiated by the prescription dose.

Note: The closer the CI value is to 1, the higher the conformity of the plan.

OAR evaluation parameters: V5, V10, V15, V20, V25, V30, V35, V40, V45, V50, D_mean_, D_max_ of heart and lungs, affected lung; cardiac substructure (AMT, LV, LA, RV, RA, LAD, LCX, RCA) V5, V10, V15, V20, V25, V30, V35, V40, V45, V50, D_mean_, D_max_; thyroid V40, V50, D_mean_, D_max_; D_mean_ and D_max_ of spinal cord.

#### Statistical analysis

All statistical analyses were performed using IBM SPSS 27.0 (IBM Corp., Armonk, NY). Continuous variables with normal distribution were presented as mean ± standard deviation, while non-normally distributed data were analyzed using nonparametric methods. For pairwise comparisons, normally distributed variables were assessed using the paired *t*-test, whereas the paired Wilcoxon signed-rank test was applied for non-normally distributed data. A two-sided significance level of α = 0.05 was adopted, with P<0.05 considered statistically significant.

## Results

### Comparison of dosimetry of two groups of target planning

For both left-sided and right-sided breast cancer, compared with the IMN group, the non-IMN group had better PTV homogeneity index (HI) and conformity index (CI) (P<0.05, **Table 1**).

**Table 1:** Comparison of dose parameters in the target area between the two groups (mean ± standard deviation).

Breast area	HI/CI	IMN group	Non-IMN group	t	P
Left breast cancer	HI	0.14±0.04	0.08±0.02	6.380	<0.001
	CI	0.85±0.04	0.88±0.02	-2.543	0.032
Right breast cancer	HI	0.20±0.06	0.09±0.02	7.205	<0.001
	CI	0.80±0.04	0.88±0.01	-6.852	<0.001

HI/CI analysis.

Subgroup analysis: dosimetric comparison of organs at risk for left breast cancer.

### Dosimetric comparison of heart and its substructures

The dosimetric comparison of the heart is shown in **Table 2**, compared with the non-IMN group, The IMN group had higher heart D_max_, D_mean_, V20, V30 (P<0.05). No significant differences were observed in heart V5, V10, V40 (**Table 2**).

**Table 2:** Comparison of dose parameters in the heart between the two groups (mean ± standard deviation).

Variable	the IMN group	non-IMN组	t	P
D_max_(cGy)	5092.77±289.48	4629.21±319.56	4.499	0.001
D_mean_(cGy)	775.26±34.58	738.17±46.19	2.368	0.042
V5	51.80±6.74	53.87±8.35	-0.627	0.546
V10	22.12±3.19	20.33±4.00	1.313	0.222
V20	7.36±1.31	4.77±1.58	5.264	0.001
V30	2.30±0.93	1.75±0.86	2.986	0.015
V40	0.45±0.32	0.39±0.38	0.654	0.530

Left-sided breast cancer analysis.

The dosimetric comparison of the heart sub-structures is shown in **Tables 3**–**10**. As shown in **Table 3**, the IMN group had lower left ventricle D_mean_ and V10 than the non-IMN group (P< 0.05), no significant differences were observed in left ventricle D_max_, V20, V30 (**Table 3**). **Table 4** shows that the IMN group had higher left atrium V5 (P< 0.05), but there are no significant differences in left atrium D_max_, D_mean_ (**Table 4**). For the right ventricle, The IMN group showed higher D_max_, D_mean_, V20, V30, V40.And lower right ventricle V5 than the non-IMN group (P< 0.05, **Table 5**). For the right atrium (RA), as shown in **Table 6**, the D_mean_ was significantly higher in the IMN group compared to the non-IMN group, with a statistically significant difference (P<0.05). No significant differences were observed in D_max_, V5, or V10 (**Table 6**). For the anterior myocardial territory (AMT), the comparative analysis (**Table 7**) revealed that D_max_ and V20 were significantly higher in the IMN group than in the non-IMN group, whereas V5 and V10 were significantly lower in the IMN group (P<0.05). No significant differences were found in D_mean_, V30, or V40 (P > 0.05, **Table 7**). For the left anterior descending artery (LAD), the results (**Table 8**) demonstrated no significant differences in D_max_, D_mean_, V5, V10, V20 or V30 (**Table 8**). For the left circumflex artery (LCX), no significant differences were observed in D_max_ or D_mean_ (**Table 9**). For the right coronary artery (RCA), D_max_ and D_mean_ were significantly higher in the IMN group than in the non-IMN group (P<0.05), whereas no significant differences were found in V5 or V10 (**Table 10**).

**Table 3:** Comparison of dose parameters in the left ventricle between the two groups (mean ± standard deviation).

Variable	the IMN group	the non-IMN group	t/w	P
D_max_(cGy)	4295.60 (3730.60, 4571.63)	4405.75 (4181.13, 4831.10)	1.89	0.059
D_mean_(cGy)	707.61±141.28	825.94±141.46	-4.498	0.001
V5	52.11 (33.90, 54.44)	64.65 (37.90, 69.16)	2.09	0.037
V10	17.30±5.53	21.50±5.99	-2.337	0.044
V20	6.89±4.17	8.19±3.13	-1.676	0.128
V30	2.15±2.03	3.00±1.94	-2.266	0.050
V40	0.08 (0, 0.28)	0.18 (0.02, 1.42)	2.033	0.042

Left-sided breast cancer analysis.

**Table 4:** Comparison of dose parameters in the left atrium between the two groups (mean ± standard deviation).

Variable	the IMN group	the non-IMN group	t	P
D_max_(cGy)	667.12±181.00	820.18±211.49	1.967	0.081
D_mean_(cGy)	363.11±66.05	313.57±48.34	1.764	0.112
V5	20.08±15.80	4.60±5.22	2.482	0.035

Left-sided breast cancer analysis.

**Table 5:** Comparison of dose parameters in the right ventricle between the two groups (mean ± standard deviation).

Variable	the IMN group	the non-IMN group	t	P
D_max_(cGy)	4361.94±681.98	2894.90±783.81	5.537	<0.001
D_mean_(cGy)	958.69±182.16	833.35±133.78	2.804	0.021
V5	68.49±16.35	77.02±14.91	-2.488	0.035
V10	34.92±12.67	28.58±13.85	1.572	0.150
V20	9.48±4.80	1.51±1.79	5.541	<0.001
V30	1.91±1.57	0.16±0.39	3.823	0.004
V40	0.30±0.34	0.00±0.01	2.785	0.021

Left-sided breast cancer analysis.

**Table 6:** Comparison of dose parameters in the right atrium between the two groups (mean ± standard deviation).

Variable	the IMN group	the non-IMN group	t	P
D_max_(cGy)	1590.92±433.06	1323.39±358.45	2.053	0.070
D_mean_(cGy)	607.45±115.44	527.89±125.13	2.591	0.029
V5	51.84±12.71	43.87±17.17	1.775	0.110
V10	15.40±13.20	8.40±9.54	1.591	0.146

Left-sided breast cancer analysis.

**Table 7:** Comparison of dose parameters in the anterior myocardial territory between the two groups (mean ± standard deviation).

Variable	the IMN group	the non-IMN group	t	P
D_max_(cGy)	5113.06±304.89	4681.78±314.07	3.794	0.004
D_mean_(cGy)	1364.88±153.97	1333.60±157.42	0.718	0.491
V5	71.66±6.10	79.17±10.31	-3.616	0.006
V10	49.08±6.15	55.42±7.31	-2.848	0.019
V20	27.13±6.41	20.22±5.90	3.487	0.007
V30	10.20±3.75	8.35±3.90	1.837	0.099
V40	2.05±1.37	1.92±1.86	0.269	0.794

Left-sided breast cancer analysis.

**Table 8:** Comparison of dose parameters in the left anterior descending artery between the two groups (mean ± standard deviation).

Variable	the IMN group	the non-IMN group	t	P
D_max_(cGy)	3896.30 (3348.68, 4641.38)	4143.95 (3479.60, 4480.95)	-0.63	0.285
D_mean_(cGy)	1520.64±455.13	1599.44±590.16	-1.064	0.315
V5	78.76±19.45	83.63±16.48	-0.639	0.539
V10	52.29±21.46	54.50±19.74	-0.384	0.710
V20	32.46±14.88	31.87±16.85	0.232	0.822
V30	12.76±12.10	16.32±17.66	-1.094	0.303

**Table 9:** Comparison of dose parameters in the left circumflex artery between the two groups (mean ± standard deviation).

Variable	the IMN group	the non-IMN group	t	P
D_max_(cGy)	696.53±237.22	681.30±195.94	0.163	0.874
D_mean_(cGy)	343.71±58.63	388.77±98.21	-1.506	0.166

**Table 10:** Comparison of dose parameters in the right coronary artery between the two groups (mean ± standard deviation).

Variable	the IMN group	the non-IMN group	t	P
D_max_(cGy)	1809.33±479.98	1394.45±397.88	2.823	0.020
D_mean_(cGy)	1000.86±365.71	815.29±250.71	2.383	0.041
V5	83.21±22.23	82.36±25.03	0.189	0.855
V10	40.94±29.61	25.52±28.72	1.851	0.097

Left-sided breast cancer analysis.

### Comparison of contralateral breast tissue dosimetry

According to the data in **Table 11**, there was no significant difference in D_max_ and D_mean_ between the two groups (**Table 11**).

**Table 11:** Comparison of dose parameters in the bilateral lungs between the two groups (mean ± standard deviation).

Variable	the IMN group	the non-IMN group	t	P
D_max_(cGy)	1108.82±82.89	1027.00±145.61	2.168	0.058
D_mean_(cGy)	374.38±66.24	370.66±528.78	0.359	0.728

Left-sided breast cancer analysis.

Subgroup analysis: dosimetric comparison of organs at risk for right breast cancer.

### Comparison of heart and its substructures

As shown in **Table 12**, the IMN group exhibited significantly higher cardiac D_max_ and V10 compared to the non-IMN group, with statistically significant differences (P< 0.05). In the IMN group, cardiac D_mean_ was higher and V5 was lower than in the non-IMN group, though these differences were not statistically significant (**Table 12**).

**Table 12:** Comparison of dose parameters in the left lungs between the two groups (mean ± standard deviation).

Variable	the IMN group	the non-IMN group	t	P
D_max_(cGy)	2175.51±682.29	1421.71±420.21	3.362	0.008
D_mean_(cGy)	352.57±70.23	351.48±65.75	0.043	0.966
V5	16.93±7.48	19.65±9.69	-0.922	0.380
V10	5.53±4.15	2.20±2.24	2.470	0.036

Right-sided breast cancer analysis.

Comparison of cardiac substructure dosimetry as shown in **Table 13**, the IMN group demonstrated significantly higher precordial D_max_ than the non-IMN group (P< 0.05). Conversely, the left circumflex artery D_max_ was significantly lower in the IMN group (P< 0.05). No statistically significant differences were observed between the two groups in precordial D_mean_, LCX D_mean_, LAD D_max_/D_mean_, or RCA D_max_/D_mean_ (**Table 13**).

**Table 13:** Comparison of thyroid dosimetric parameters between the two groups (mean ± standard deviation).

Variable	the IMN group	the non-IMN group	t	P
LV D_max_ (cGy)	475.29±260.01	486.87±119.64	-0.168	0.870
LV D_mean_ (cGy)	188.64±33.77	220.00±57.06	-1.973	0.080
LA D_max_ (cGy)	958.13±541.67	728.69±239.61	1.124	0.290
LA D_mean_ (cGy)	267.59±103.47	261.78±74.75	0.147	0.887
RV D_max_ (cGy)	1086.45±329.06	978.39±367.98	0.967	0.359
RVD_mean_ (cGy)	331.14±69.65	343.75±59.07	-0.730	0.484
RA D_max_ (cGy)	1792.02±656.53	1210.88±253.82	2.515	0.033
RA D_mean_ (cGy)	596.53±219.94	590.30±145.65	0.077	0.940
AMT D_max_ (cGy)	2306.66±781.38	1430.50±427.64	3.818	0.004
AMT D_mean_ (cGy)	566.61±130.80	490.68±133.16	1.908	0.089
LAD D_max_ (cGy)	527.29±234.03	529.72±112.02	-0.037	0.971
LAD D_mean_ (cGy)	284.12±75.17	303.07±65.07	-0.734	0.482
LCX D_max_ (cGy)	228.02±23.71	358.41±145.93	-2.955	0.016
LCX D_mean_ (cGy)	171.35 (159.53, 185.18)	183.45 (163.75, 242.40)	-1.682	0.093
RCA D_max_ (cGy)	1408.30±579.07	1148.520	1.554	0.155
RCA D_mean_ (cGy)	597.22±273.07	664.36±226.14	-1.165	0.274

Right-sided breast cancer analysis.LV, left ventricle; LA, left atrium; RV, right ventricle; RA, right atrium; AMT, anterior myocardial territory; LAD, left anterior descending artery; LCA= left circumflex artery; RCA, right coronary artery.

### Comparison of contralateral breast tissue dosimetry

**Table 14** demonstrated statistically significant differences in contralateral breast D_max_ and D_mean_ of the healthy breast tissue between the two groups (P< 0.05, **Table 14**).

**Table 14:** Comparison of dosimetric parameters of the contralateral breast in the two groups (mean ± standard deviation).

Variable	the IMN group	the non-IMN group	t	p
D_max_(cGy)	1148.08±99.99	1006.28±83.01	3.341	0.009
D_mean_(cGy)	354.04±97.41	301.6274.95	4.415	0.002

Right-sided breast cancer analysis.

## Discussion

In China, breast cancer represents the second most common malignancy among women and ranks fifth in cancer-related mortality (9). Advances in multimodal treatment strategies have significantly improved long-term survival outcomes for breast cancer patients. Radiotherapy remains a critical component of adjuvant therapy following breast-conserving surgery, with only a select minority of patients eligible for postoperative radiotherapy omission. However, optimal target delineation for adjuvant irradiation remains controversial, particularly regarding inclusion of the internal mammary lymph node (IMN) region. Historically, extended radical mastectomy with IMN dissection was associated with prolonged operative duration, no survival benefit, and increased postoperative morbidity (10). Consequently, routine IMN dissection has been largely abandoned in clinical practice. This shift has renewed interest in internal mammary nodal irradiation (IMNI) as an alternative strategy, prompting ongoing debate among clinicians and researchers. Emerging evidence demonstrates that internal mammary nodal irradiation (IMNI) confers survival benefits for select breast cancer patients (11–14), with particularly pronounced advantages observed in high-risk populations (15). However, these oncologic benefits must be carefully weighed against potential cardiopulmonary toxicity. IMNI increases radiation exposure to critical organs, elevating risks of clinically significant heart and lung injuries that may compromise quality of life and overall survival. Existing data indicate breast cancer radiotherapy recipients face a 1.5- to 3-fold increased risk of cardiovascular complications compared to non-irradiated patients (8). As highlighted by He et al. (16), the long-term survival advantage of radiotherapy may be partially attenuated by late cardiovascular sequelae, including radiation-induced heart disease. This risk-benefit paradox underscores the need for precise patient selection and advanced radiation techniques to optimize therapeutic outcomes.The heart is classified as a late-responding tissue, with clinical symptoms typically emerging 6–12 months post-radiation, while severe late-stage manifestations often develop over >10 years. The pathophysiological cascade of radiation-induced cardiotoxicity involves endothelial damage causing capillary stenosis and reduced vascular-to-cardiomyocyte ratios, culminating in cardiomyocyte death (17). Radiation triggers inflammatory cytokine production and promotes smooth muscle cell differentiation into myofibroblasts, which deposit excessive collagen that replaces both myocardial interstitium and necrotic cardiomyocytes. Valvular pathology demonstrates characteristic fibrotic thickening of the tips and leaflets, frequently with calcification (18). Histopathological analysis of post-radiation aortic valves revealed dose-dependent depletion of cellular components with concomitant collagen/fibrous tissue accumulation compared to non-irradiated controls, establishing a clear dose-response relationship for valvular fibrosis (19).

Studies have demonstrated distinct patterns of radiation-associated cardiac injury. Darby et al. (20) identified the anterior wall of the left ventricle as the most frequently affected site. Similarly, Correa et al. (21) reported preferential involvement of the left anterior descending (LAD) coronary artery in radiation-induced coronary injury, with Nilsson et al. (22) further localizing the mid-to-distal LAD and distal diagonal branches as high-risk regions in left-sided breast cancer radiotherapy.

Long-term clinical data from landmark studies reveal differential cardiovascular risks associated with radiotherapy approaches: breast-conserving surgery followed by radiation confers a 1.1% absolute increase in 15-year cardiovascular event risk compared to non-irradiated controls, while post-mastectomy radiotherapy elevates this risk by 5.6% (23). Radiation-associated atherosclerosis typically manifests 10–15 years post-treatment, predominantly affecting the LAD and right coronary arteries.

The heart’s dynamic nature poses unique challenges – its continuous motion limits accurate dosimetric assessment, and its volumetric size creates an inherent conflict between optimal target coverage and cardiac dose minimization. Current evidence suggests that focused protection of cardiac substructures (left ventricle [LV], LAD, and anterior myocardial territory [AMT]) as organs at risk may represent a more achievable optimization strategy.

Tan (24) first proposed the concept of AMT, which encompasses the anterior myocardial wall, including the left and right coronary arteries, the LAD, and adjacent myocardial tissue. A comparative study evaluating the impact of contouring the whole heart versus AMT as an OAR on normal tissue dose distribution demonstrated that using AMT as an OAR reduced the mean dose to the heart, LV, AMT, right lung, and right breast. Thus, the study recommended replacing the whole heart with AMT as an OAR in intensity-modulated radiation therapy (IMRT) planning. Wang et al. (25) confirmed that delineating the LV as an OAR in IMRT for left-sided breast cancer significantly reduced cardiac irradiation doses. Furthermore, adding the LV as an additional OAR for plan evaluation, beyond the whole heart, provided greater advantages in minimizing long-term radiation-induced cardiac injury. Zhou (26) reported that for left-sided breast cancer patients undergoing deep inspiration breath-hold (DIBH) during IMRT (including internal mammary node irradiation), the mean dose (Dmean), maximum dose (Dmax), and dose-volume parameters (V5–V30) of the heart and LAD were significantly reduced (P< 0.05). A meta-analysis (27) of 11 studies involving 13,181 breast cancer patients found that prophylactic internal mammary lymph node irradiation (IMNI) improved disease-free survival and may provide an overall survival benefit for high-risk stage III (N+, T3–4) patients. Zhu et al. (28) observed that the IMNI group had slightly higher cardiac Dmean, V30, and V40 than the non-IMNI group (P< 0.05). Although cardiac doses increased, they remained within acceptable limits, with no significant short-term decline in cardiac function or quality of life. Our study yielded similar findings. In left-sided breast cancer patients, the IMNI group exhibited higher cardiac Dmax and Dmean than the non-IMNI group (P< 0.05). However, the IMNI group had a lower LV Dmean. Additionally, the IMNI group showed higher Dmax and Dmean in the right ventricle, right atrium, anterior myocardial territory, and right coronary artery (P< 0.05). No statistically significant differences were observed in LV Dmax, left atrial Dmax/Dmean, right atrial Dmax, anterior myocardial territory Dmean, LAD Dmax/Dmean, or left circumflex artery Dmax/Dmean. These findings reinforce the importance of adhering to guideline recommendations for IMNI.Cardio-oncology is an emerging discipline, and cardiac irradiation dose remains a critical research topic in thoracic radiotherapy. Clinical practice conventionally employs mean heart dose (MHD) for cardiac risk assessment; however, accumulating evidence demonstrates poor correlation between MHD and subsequent cardiovascular events, with limited capacity to predict cardiac injury severity. Notably, radiation exposure to specific cardiac substructures - particularly the LV and coronary arteries - shows significantly stronger associations with cardiovascular risk (29). This spatial dependence manifests clinically as distinct injury patterns corresponding to different irradiated anatomical regions and dose distributions. Taylor et al. (30) established that during radiotherapy for left-sided breast cancer, the LV apex receives maximal radiation exposure, correlating with a hierarchical risk gradient for myocardial ischemia: highest at the apex, followed by the interventricular septum and anterior LV wall. Dose distribution patterns demonstrate significant dependence on three key factors: tumor laterality, radiation beam geometry, and IMNI inclusion. Notably, the study revealed two clinically important scenarios: patients with comparable MHD may exhibit substantially different cardiac substructure doses, and certain cases with acceptable MHD may still deliver critical doses to vulnerable substructures. Consequently, using mean heart dose (MHD) as the sole dosimetric parameter proves inadequate for precise cardiovascular risk stratification. In the current precision medicine paradigm, comprehensive plan evaluation must integrate both cardiac substructure dose constraints and MHD to achieve optimal cardioprotection in radiation oncology practice.

Computed tomography (CT) slice thickness determines the spatial resolution of images along the z-axis. A smaller slice thickness yields higher z-axis resolution and reduces partial volume effects, thereby enabling more accurate representation of the surface and internal anatomical structures of the scanned object (31). Zhao et al. (32) reported that CT slice thickness significantly influences dose distribution to the target in CyberKnife radiotherapy, with this effect being particularly pronounced for smaller tumors. Luo et al. (33) found that as CT slice thickness increases, the volumetric error in contoured structures increases significantly, while the planned conformity index (CI) systematically decreases. For instance, at a 6 mm slice thickness, although the total heart volume decreased by only 3.2%, V^30^ and V^40^ increased significantly by 18.4% and 46.6%, respectively. In this study, a slice thickness of 5 mm was used for scanning. This implies that if a treatment plan designed on 2 mm thin-slice images is directly mapped onto 5 mm slice images for dose calculation, it may erroneously indicate increased radiation exposure to the heart. This potential limitation has been acknowledged and taken into consideration in the present study.

IMRT dose calculation is performed on a three-dimensional grid, the size of which is typically matched to the imaging resolution. When the z-axis resolution is only 5 mm, the dose gradient in this direction cannot be accurately modeled. For precision techniques such as IMRT, which rely on steep dose gradients to spare organs at risk, insufficient z-axis resolution may lead to “stair-step” artifacts, thereby compromising its physical advantages and resulting in discrepancies between calculated and delivered dose distributions. Srivastava et al. (34) also confirmed that thinner CT slices improve volumetric accuracy—particularly in the delineation of small structures—enhance target dose coverage, and help improve the homogeneity index (HI) and conformity index (CI), thus ensuring overall treatment plan quality.

Given that all patient data in this study were based on 5 mm slice images, the current analysis has precluded errors arising from inconsistencies in CT slice thickness. In subsequent research, the team will systematically compare various dosimetric and planning metrics across different slice thicknesses to further validate their impact.

Several limitations should be considered when interpreting our findings: (1) The retrospective design and relatively small sample size (n=20) may introduce selection bias; (2) Dosimetric analysis was performed exclusively on simulation CT images without clinical follow-up data to correlate with long-term outcomes; (3) The study was confined to static eight-field IMRT planning without comparison to alternative techniques such as volumetric modulated arc therapy (VMAT) or other multi-field irradiation approaches. (4) all cases in this study were based on 5 mm slice thickness images, and the influence of this factor on the indexes of heart and other organs at risk in radiotherapy plan was not considered due to the thickness of CT image. These methodological constraints highlight the need for prospective validation studies with larger cohorts and comprehensive clinical follow-up. With advancements in cardio-oncology, we have implemented routine cardiac substructure delineation in clinical practice and enhanced patient follow-up protocols to systematically collect survival outcomes, quality-of-life metrics, and cardiovascular events. Our ongoing efforts include expanding the cohort size to strengthen the validity of our findings. Furthermore, we plan to implement prospective clinical trials to optimize radiotherapy techniques, with particular emphasis on reducing radiation exposure to critical cardiac substructures in post-lumpectomy breast cancer patients.

## Conclusion

1. Dosimetric analysis confirms that internal mammary node (IMN) irradiation using an 8-field IMRT technique significantly elevates mean heart dose and exposure to critical cardiac substructures in left-sided breast cancer (P< 0.05), thereby increasing the potential for long-term cardiovascular toxicity. For right-sided disease, however, IMN inclusion did not significantly alter mean heart dose (P > 0.05) or dose to the left anterior descending artery. This marked lateral disparity necessitates a tailored approach to clinical decision-making, where the competing risks of locoregional recurrence and potential cardiotoxicity must be carefully balanced. For left-sided patients, this risk-benefit assessment should strongly favor the adoption of advanced cardiac-sparing techniques.
2. Our findings challenge the adequacy of current planning metrics, which predominantly rely on global heart dose-volume parameters. These metrics may fail to sufficiently capture the risk of radiation-induced cardiotoxicity, as significant exposures to critical substructures can occur even when whole-heart doses appear acceptable. We therefore propose a paradigm shift toward substructure-informed planning, which mandates the delineation of cardiac substructures (e.g., ventricles, coronary arteries) as discrete organs at risk and the systematic integration of their dose constraints into both optimization and quality assurance protocols. This strategy is pivotal for enhancing the therapeutic ratio by proactively minimizing late cardiovascular complications.
3. In conclusion, while adherence to established guidelines for IMN irradiation remains paramount for ensuring optimal oncologic outcomes, our study underscores that this must be coupled with rigorous cardiac protection. The implementation of advanced radiotherapy techniques is strongly advocated to mitigate cardiac risks, particularly for left-sided breast cancer, representing an essential step toward achieving safer and more personalized radiotherapy.

## Data Availability

The original contributions presented in the study are included in the article/supplementary material, further inquiries can be directed to the corresponding author/s.

## References

[1] Bray F Laversanne M Sung H Ferlay J Siegel R L Soerjomataram I . Global cancer statistics 2022: GLOBOCAN estimates of incidence and mortality worldwide for 36 cancers in 185 countries. CA Cancer J Clin. (2024) 74:229–63. doi: 10.3322/caac.21834, PMID: 38572751

[2] Early Breast Cancer Trialists' Collaborative Group (EBCTCG) Darby S McGale P Correa C Taylor C Arriagada R . Effect of radiotherapy after breast-conserving surgery on 10-year recurrence and 15-year breast cancer death: meta-analysis of individual patient data for 10 801 women in 17 randomised trials. Lancet. (2011) 378:1707–16. doi: 10.1016/S0140-6736(11)61629-2, PMID: 22019144 PMC3254252

[3] Stemmer SM Rizel S Hardan I Adamo A Neumann A Goffman J . The role of irradiation of the internal mammary lymph nodes in high-risk stage II to IIIA breast cancer patients after high-dose chemotherapy: a prospective sequential nonrandomized study. J Clin Oncol. (2003) 21:2713–8. doi: 10.1200/JCO.2003.09.096, PMID: 12860949

[4] Darby SC Ewertz M McGale P Bennet AM Blom-Goldman U Brønnum D . Risk of ischemic heart disease in women after radiotherapy for breast cancer. N Engl J Med. (2013) 368:987–98. doi: 10.1056/NEJMoa1209825, PMID: 23484825

[5] Darby SC McGale P Taylor CW Peto R . Long-term mortality from heart disease and lung cancer after radiotherapy for early breast cancer: prospective cohort study of about 300,000 women in US SEER cancer registries. Lancet Oncol. (2005) 6:557–65. doi: 10.1016/S1470-2045(05)70251-5, PMID: 16054566

[6] Taylor CW Nisbet A McGale P Darby SC . Cardiac exposures in breast cancer radiotherapy: 1950s-1990s. Int J Radiat Oncol Biol Phys. (2007) 69:1484–95. doi: 10.1016/j.ijrobp.2007.05.034, PMID: 18035211

[7] Schultz-Hector S Trott KR . Radiation-induced cardiovascular diseases: is the epidemiologic evidence compatible with the radiobiologic data? Int J Radiat Oncol Biol Phys. (2007) 67:10–8. doi: 10.1016/j.ijrobp.2006.08.071, PMID: 17189062

[8] Clarke M Collins R Darby S Davies C Elphinstone P Evans V . Effects of radiotherapy and of differences in the extent of surgery for early breast cancer on local recurrence and 15-year survival: an overview of the randomised trials. Lancet. (2005) 366:2087–106. doi: 10.1016/S0140-6736(05)67887-7, PMID: 16360786

[9] Zheng RS Chen R Han BF Wang SM Li L Sun KX . Analysis of cancer incidence and mortality in China, 2022. Chinese Journal of Oncology. (2024) 46(3):221–231. doi: 10.3760/cma.j.cn112152-20240119-00035, PMID: 38468501

[10] Veronesi U Marubini E Mariani L Valagussa P Zucali R . The dissection of internal mammary nodes does not improve the survival of breast cancer patients. 30-year results of a randomised trial. Eur J Cancer. (1999) 35:1320–5. doi: 10.1016/S0959-8049(99)00133-1, PMID: 10658521

[11] Jia S Liu Z Zhang J Zhao C Zhu L Kong J . Can internal mammary lymph nodes irradiation bring survival benefits for breast cancer patients? A systematic review and meta-analysis of 12,705 patients in 12 studies. Radiat Oncol. (2021) 16:42. doi: 10.1186/s13014-021-01772-y, PMID: 33622345 PMC7903795

[12] Luo J Jin K Chen X Wang X Yang Z Zhang L . Internal mammary node irradiation (IMNI) improves survival outcome for patients with clinical stage II-III breast cancer after preoperative systemic therapy. Int J Radiat Oncol Biol Phys. (2019) 103:895–904. doi: 10.1016/j.ijrobp.2018.11.003, PMID: 30439485

[13] Poortmans PM Weltens C Fortpied C Kirkove C Peignaux-Casasnovas K Budach V . Internal mammary and medial supraclavicular lymph node chain irradiation in stage I-III breast cancer (EORTC 22922/10925): 15-year results of a randomised, phase 3 trial. Lancet Oncol. (2020) 21:1602–10. doi: 10.1016/S1470-2045(20)30472-1, PMID: 33152277

[14] Wang X Luo J Jin K Chen X Zhang L Meng J . Internal mammary node irradiation improves 8-year survival in breast cancer patients: results from a retrospective cohort study in real-world setting. Breast Cancer. (2020) 27:252–60. doi: 10.1007/s12282-019-01015-9, PMID: 31696449

[15] Korzets Y Levitas D Grubstein A Corn BW Amir E Goldvaser H . Efficacy and safety of the addition of internal mammary irradiation to standard adjuvant radiation in early-stage breast cancer: A systematic review and meta-analysis. Curr Oncol. (2022) 29:6657–73. doi: 10.3390/curroncol29090523, PMID: 36135092 PMC9497563

[16] He J Wang S Liu H Duan C Zhang H Wen F . Competing risk analysis of cardiovascular death in breast cancer: evidence from the SEER database. Transl Cancer Res. (2023) 12:3591–603. doi: 10.21037/tcr-23-1163, PMID: 38192997 PMC10774043

[17] Heidenreich PA Hancock SL Vagelos RH Lee BK Schnittger I . Diastolic dysfunction after mediastinal irradiation. Am Heart J. (2005) 150:977–82. doi: 10.1016/j.ahj.2004.12.026, PMID: 16290974

[18] Belzile-Dugas E Eisenberg MJ . Radiation-induced cardiovascular disease: review of an underrecognized pathology. J Am Heart Assoc. (2021) 10:e021686. doi: 10.1161/JAHA.121.021686, PMID: 34482706 PMC8649542

[19] Van Rijswijk JW Farag ES Bouten CVC de Boer OJ van der Wal A de Mol B . Fibrotic aortic valve disease after radiotherapy: an immunohistochemical study in breast cancer and lymphoma patients. Cardiovasc Pathol. (2020) 45:107176. doi: 10.1016/j.carpath.2019.107176, PMID: 31837504

[20] Darby SC Cutter DJ Boerma M Constine LS Fajardo LF Kodama K . Radiation-related heart disease: current knowledge and future prospects. Int J Radiat Oncol Biol Phys. (2010) 76:656–65. doi: 10.1016/j.ijrobp.2009.09.064, PMID: 20159360 PMC3910096

[21] Correa CR Litt HI Hwang WT Ferrari VA Solin LJ Harris EE . Coronary artery findings after left-sided compared with right-sided radiation treatment for early-stage breast cancer. J Clin Oncol. (2007) 25:3031–7. doi: 10.1200/JCO.2006.08.6595, PMID: 17634481

[22] Nilsson G Holmberg L Garmo H Duvernoy O Sjögren I Lagerqvist B . Distribution of coronary artery stenosis after radiation for breast cancer. J Clin Oncol. (2012) 30:380–6. doi: 10.1200/JCO.2011.34.5900, PMID: 22203772

[23] Yilmaz M Turk E Sana MK Olafimihan A Uygun I Shoura S . Cardiovascular outcomes associated with exposure to radiation therapy in thoracic Malignancies: an insight study using the national inpatient database. Cureus. (2023) 15:e47113. doi: 10.7759/cureus.47113, PMID: 38021583 PMC10647132

[24] Tan W Liu D Xue C Xu J Li B Chen Z . Anterior myocardial territory may replace the heart as organ at risk in intensity-modulated radiotherapy for left-sided breast cancer. Int J Radiat Oncol Biol Phys. (2012) 82:1689–97. doi: 10.1016/j.ijrobp.2011.03.009, PMID: 21621342

[25] Wang XH Liu D Wang JY Tan WY Hu DS . Cardiac Protection by Considering Left Ventricle as an Organ at Risk in Postoperative Radiotherapy for Left-Sided Breast-Conserving Surgery. Cancer Research on Prevention and Treatment. (2012) 39(6):731–4. doi: 10.3971/j.issn.10008578.2012.06.031

[26] Zhou YC Sun XL Wu H Sun N Li W Han Y . Dosimetric impact of deep inspiration breath-hold technique on cardiac structures during internal mammary node-inclusive intensity-modulated radiotherapy for left-sided postmastectomy breast cancer. Chinese Journal of Radiological Medicine and Protection. (2023) 43(12):979–85. doi: 10.3760/cma.j.cn112271-20230626-00209

[27] Jia SC Liu ZK Zhang J Zhao CG Zhou LY Kong J . A Meta-analysis of Prophylactic Internal Mammary Lymph Node Irradiation in Breast Cancer. Chinese Journal of Radiation Oncology. (2021) 30(9):903–9. doi: 10.3760/cma.j.cn113030-20201022-00508

[28] Zhu QW Cui JJ Zhang ZH Yang YG Ge BB Liu Y . Dosimetric Analysis of Cardiac Exposure and Quality-of-Life Assessment Following Internal Mammary Nodal Irradiation in Postoperative Left-Sided Breast Cancer Patients. Journal of International Oncology. (2023) 50(1):17–21. doi: 10.3760/cma.j.cn371439-20221123-00003

[29] Jacob S Camilleri J Derreumaux S Walker V Lairez O Lapeyre M . Is mean heart dose a relevant surrogate parameter of left ventricle and coronary arteries exposure during breast cancer radiotherapy: a dosimetric evaluation based on individually-determined radiation dose (BACCARAT study). Radiat Oncol. (2019) 14:29. doi: 10.1186/s13014-019-1234-z, PMID: 30732640 PMC6367844

[30] Taylor C McGale P Brønnum D Correa C Cutter D Duane FK . Cardiac structure injury after radiotherapy for breast cancer: cross-sectional study with individual patient data. J Clin Oncol. (2018) 36:2288–96. doi: 10.1200/JCO.2017.77.6351, PMID: 29791285 PMC6067799

[31] Chen GL Zhao R Li JJ Li S . Exploration of related helical CT positioning conditions in CyberKnife radiotherapy. China Medical Devices. (2018) 33(5):90–2. doi: 10.3969/j.issn.1674-1633.2018.05.024

[32] Zhao R Cao YS Gao XX Wang ZY Li S Tian ZZ . Influence of CT slice thickness on target dose distribution in CyberKnife radiotherapy based on chest phantom. Chinese Journal of Medical Physics. (2023) 40(6):667–71. doi: 10.3969/j.issn.1005-202X.2023.06.002

[33] Luo H He Y Jin F Yang D Liu X Ran X . Impact of CT slice thickness on volume and dose evaluation during thoracic cancer radiotherapy. Cancer Manag Res. (2018) 10:3679–86. doi: 10.2147/CMAR.S174240, PMID: 30288099 PMC6159785

[34] Srivastava SP Cheng CW Das IJ . The effect of slice thickness on target and organs at risk volumes, dosimetric coverage and radiobiological impact in IMRT planning. Clin Transl Oncol. (2016) 18:469–79. doi: 10.1007/s12094-015-1390-z, PMID: 26311077

